# Introducing site-specific cysteines into nanobodies for mercury labelling allows *de novo* phasing of their crystal structures

**DOI:** 10.1107/S2059798317013171

**Published:** 2017-09-27

**Authors:** Simon Boje Hansen, Nick Stub Laursen, Gregers Rom Andersen, Kasper R. Andersen

**Affiliations:** a Department of Molecular Biology and Genetics, Aarhus University, Gustav Wieds Vej 10C, 8000 Aarhus, Denmark

**Keywords:** phasing, nanobodies, mercury, single-wavelength anomalous dispersion, SAD, single isomorphous replacement with anomalous signal, SIRAS

## Abstract

The generation of high-quality protein crystals and the loss of phase information during an X-ray crystallography diffraction experiment represent the major bottlenecks in the determination of novel protein structures. A generic method for introducing Hg atoms into any crystal independent of the presence of free cysteines in the target protein could considerably facilitate the process of obtaining unbiased experimental phases. Nanobodies (single-domain anti­bodies) have recently been shown to promote the crystallization and structure determination of flexible proteins and complexes. To extend the usability of nanobodies for crystallographic work, variants of the Nb36 nanobody with a single free cysteine at one of four framework-residue positions were developed. These cysteines could be labelled with fluorophores or Hg. For one cysteine variant (Nb36-C85) two nanobody structures were experimentally phased using single-wavelength anomalous dispersion (SAD) and single isomorphous replacement with anomalous signal (SIRAS), taking advantage of radiation-induced changes in Cys–Hg bonding. Importantly, Hg labelling influenced neither the interaction of Nb36 with its antigen complement C5 nor its structure. The results suggest that Cys–Hg-labelled nanobodies may become efficient tools for obtaining *de novo* phase information during the structure determination of nanobody–protein complexes.

## Abstract 2

Nanobodies are used as crystallization chaperones and here site-specific mercury labelling of nanobodies is shown as a new tool for phasing.

## 1. Introduction   

The production of well diffracting protein crystals is a major challenge in macromolecular X-ray crystallography. Large multi-domain proteins and membrane proteins are inherently difficult to crystallize owing to conformational heterogeneity and the lack of suitable surface chemistry that allows the formation of a crystal lattice. Crystallization chaperones are auxiliary proteins that increase the chance of crystallization by reducing conformational flexibility and providing well ordered surfaces to form crystal lattice contacts. Monoclonal antibody Fab fragments derived from IgG are the most widely used chaperones (Uysal *et al.*, 2009 ▸) and may simultaneously provide phase information for structure determination by molecular replacement. Several alternative chaperones have been developed, including DARPins, single-chain variable fragments and nanobodies (Nbs; Pardon *et al.*, 2014 ▸). Nbs are derived from natural llama heavy-chain antibodies that are devoid of a light chain and in which the heavy-chain variable domain (V_H_H) exclusively mediates the interaction with the antigen (Muyldermans, 2013 ▸). The V_H_H domain is structurally similar to the IgG V_H_ domain, with three complementary-determining regions (CDRs) that are responsible for antigen binding. In contrast to Fab fragments, which must be expressed in mammalian or insect cells, Nbs are easy to express and manipulate in *Escherichia coli*. As a result of their favourable characteristics Nbs are also increasingly being used in imaging, where they can be labelled with GFP or fluorescent dyes (Chakravarty *et al.*, 2014 ▸; Rothbauer *et al.*, 2006 ▸). Covalent attachment of fluorescent dyes to Nbs has proven to be effective using NHS ester, isothiocyanate or maleimide functional groups, with maleimide labelling being superior to NHS ester dyes when comparing background staining in cell-permeabilizing imaging (Pleiner *et al.*, 2015 ▸; Röder *et al.*, 2017 ▸). NHS esters and isothiocyanates react readily with N-terminal and lysine amines, while maleimide reacts specifically with cysteine thiols in the pH range 6.5–7.5.

For crystallization purposes, the antigen can be screened in complex with each identified Nb or in combinations with more than one Nb to increase the probability of crystallization (Zhang *et al.*, 2015 ▸). However, owing to the small size of Nbs the phase information obtained from a single Nb by molecular replacement is limited and may not be sufficient to provide initial phases for a Nb-complex structure. We rationalized that generating heavy-atom-labelled Nbs would allow one to not only utilize Nbs as crystallization chaperones but also provide an easy approach for experimental phasing. Incorporation of selenomethionines is a successful route for introducing anomalous scatterers into proteins for subsequent experimental phasing, but this approach is very challenging for proteins expressed in eukaryotic cells and is not feasible for proteins purified from natural sources. Alternatively, denser atoms (for example mercury, gold and platinum) have successfully been used as phasing labels (Pike *et al.*, 2016 ▸). One strategy uses cysteines for site-specific mercury labelling and has helped to solve the structures of both soluble and membrane proteins (Doyle *et al.*, 1996 ▸; Li *et al.*, 2015 ▸). Hg is the most successful element when it comes to forming heavy-atom derivatives of proteins and their crystals, and cysteine side chains are also by far the most frequent binding or coordinating residues for Hg atoms (Sugahara *et al.*, 2005 ▸). Hg has been extensively used for isomorphous replacement since the early days of protein crystallography, where it was used to determine the structure of haemoglobin (Green *et al.*, 1954 ▸), and inclusion of the anomalous signal from Hg for SIRAS phasing was reported in 1977 (Wood *et al.*, 1977 ▸). SAD phasing is now the standard approach for obtaining experimental phase information in macromolecular crystallography since the majority of modern synchrotrons have beamlines with tunable wavelengths and the potential problem of non-isomorphism is avoided (Rose & Wang, 2016 ▸). The collection of anomalous data from a single well diffracting Hg-substituted crystal is a relatively simple task, whereas merging anomalous data from multiple small crystals is more challenging and the anomalous signal is also even more sensitive to radiation damage than the overall reciprocal-space signal. Recently, SAD and SIRAS phasing based on data obtained by femtosecond serial crystallography for the luciferin-regenerating enzyme with a single Hg-substituted cysteine in a protein with 308 residues was reported (Yamashita *et al.*, 2015 ▸). For the same protein SAD phasing was also successful using serial synchrotron rotation crystallography (Hasegawa *et al.*, 2017 ▸). Thus, very challenging structures for which only microcrystals are available may have their structures determined rapidly if Hg atoms are present in the crystals. Hence, a generic method for introducing Hg atoms into any crystal independent of the presence of free cysteines in the target protein could greatly facilitate the process of obtaining unbiased experimental phases. Disulfide bridges in extracellular proteins can, upon partial reduction, react with Hg^2+^ and thus have a Hg atom inserted between two cysteines connected by a disulfide bridge (Sperling *et al.*, 1969 ▸), but this confers the risk of decreasing the stability of the protein and perturbation of its structure. We describe the introduction of cysteine residues at conserved framework serine positions in a Nb specific for human complement component C5 and subsequent site-specific labelling with Hg derivatives and structure determination by SAD and SIRAS. Hg incorporation did not perturb the structure of the Nb or its antigen-binding capacity. We also show that the introduced cysteines can be labelled with Alexa Fluor 488, providing a generic method for generating fluorescent Nbs for molecular imaging.

## 2. Methods   

### 2.1. Nanobody production   

Human C5 and cobra venom factor (CVF) were purified as described previously (Laursen *et al.*, 2010 ▸; Schatz-Jakobsen *et al.*, 2016 ▸). One llama (*Lama glama*) was immunized with 500 µg human C5. Total RNA was isolated from peripheral blood lymphocytes using an RNase Plus Mini Kit (Qiagen) and cDNA was generated using a SuperScript III First-Strand Kit (Invitrogen) with random hexamer primers. Nb DNA sequences were amplified by PCR and inserted into a phagemid vector designed to express Nbs as pIII fusions. The M13 phage-display Nb library was generated using the VCSM13 helper phage. For Nb selection, a microtitre plate well was coated with 1 µg C5 and was blocked after 12 h with PBS supplemented with 2% BSA. A total of 3 × 10^12^ M13 phage particles were added and allowed to bind C5 for 1 h before 15 washing steps with PBS containing 0.1% Tween 20. The remaining phage particles binding to C5 at its CVF interface (Laursen *et al.*, 2011 ▸) were eluted by adding 100 µl CVF at 1 mg ml^−1^ for 1 h. The eluted phage particles were then added to *E. coli* ER2738 cells. The enriched library was amplified and used in a second round of phage display, but this time using only 0.1 µg C5 and 3 × 10^12^ M13 phage particles. Phage particles were eluted at low pH by adding 100 µl 0.2 *M* glycine pH 2.2 for 15 min and then neutralized with 15 µl 1 *M* Tris pH 9.1 before being added to *E. coli* ER2738 cells. After two rounds of phage-display selection, single colonies were transferred to a 96-well plate format and grown for 6 h in LB medium before Nb expression was induced with 0.8 m*M* IPTG overnight at 30°C. The 96-well plate was centrifuged and 50 µl of the supernatant was transferred to an ELISA plate coated with 1 µg ml^−1^ C5 in blocking solution (PBS with 0.1% Tween 20 and 2% BSA). The ELISA plate was then washed six times in PBS with 0.1% Tween 20 before anti-E-tag-HPR antibody (Bethyl) was added at a 1:10 000 dilution. The plate was washed and developed with 3,3′,5,5′-tetramethylbenzidine, the reaction was quenched with 1 *M* HCl and the plate was read at 450 nm. Phagemids from positive clones were isolated, sequenced and subcloned for bacterial expression. DNA encoding Nb36 was cloned into a pET-22b(+) expression vector and the cysteine mutants were generated using inverse PCR. The Nb constructs contained an N-terminal PelB signal for secretion into the periplasm and a C-terminal 6×His tag. All Nbs were expressed in *E. coli* LOBSTR cells (Andersen *et al.*, 2013 ▸) grown to an optical density of ∼0.6 before expression was induced with 0.2 m*M* IPTG at 18°C overnight. Cells were opened in lysis buffer [PBS buffer supplemented with 400 m*M* NaCl and 20 m*M* imidazole and with an additional 5 m*M* β-mercaptoethanol (BME) for the cysteine mutants]. The cleared supernatant was loaded onto Ni Sepharose 6 FF affinity resin (GE Healthcare) and extensively washed before elution in lysis buffer supplemented with 400 m*M* imidazole. The Nbs were finally purified on a Superdex 75 10/300 gel-filtration column (GE Healthcare) in gel-filtration buffer [10 m*M* HEPES pH 7.6, 150 m*M* NaCl with an additional 2 m*M* dithiothreitol (DTT) for the cysteine mutants] and concentrated before labelling and crystallization.

### 2.2. Site-specific mercury labelling   

Immediately before labelling, the Nbs were transferred into nonreducing buffer (10 m*M* HEPES pH 7.6, 150 m*M* NaCl) using a PD-10 desalting column. Nbs were immediately mixed with a fivefold molar excess of *para*-chloromercuribenzoic acid (PCMB) or a tenfold molar excess of mercury(II) acetate and incubated on ice for 1 h. PCMB labelling was quenched after 1 h by the addition of a 100-fold molar excess of iodo­acetamide. To quantify the PCMB labelling efficiency, the remaining free cysteines were reacted with a tenfold molar excess of MPEG–maleimide (Sigma–Aldrich, catalogue No. 99126-64-4) on ice for 1 h. The samples were analysed by nonreducing SDS–PAGE and the ratio of Nb that had reacted with MPEG–maleimide to Nb that had reacted with PCMB was compared and quantified using *ImageJ* (Hartig, 2013 ▸).

### 2.3. Site-specific fluorescent labelling   

As for PCMB labelling, the Nbs were transferred into a nonreducing buffer (10 m*M* HEPES pH 7.6, 50 m*M* NaCl) using a PD-10 desalting column. Nbs were mixed with a 1.5-fold molar excess of Alexa Fluor 488 maleimide (Thermo Fisher) and incubated on ice for 1 h before quenching with a 100-fold molar excess of DTT. The samples were analysed by nonreducing SDS–PAGE and fluorescence detection of the resulting acrylamide gel on a Typhoon fluorescent scanner (GE Healthcare).

### 2.4. Crystallization   

Before crystallization, PCMB-labelled monomeric Nbs were separated from a minor fraction of Nb dimers on a Mono S 5/50 cation-exchange column (GE) equilibrated in 20 m*M* sodium acetate pH 5.5. The pH and salt concentration of the sample were adjusted before loading by mixing the PCMB-labelled Nb with one volume of 40 m*M* sodium acetate pH 5.5. PCMB-labelled Nbs were eluted using a linear gradient from 250 to 500 m*M* NaCl. The buffer was exchanged into gel-filtration buffer on a spin filter (Vivaspin 500 centrifugal concentrators) and the protein was concentrated to 8–10 mg ml^−1^. All crystals were formed in vapour-diffusion experiments by mixing equal volumes of protein and reservoir solutions. Crystals of nonmodified Nb36 (Nb36-Nat1 and Nb36-Nat2) were obtained by vapour diffusion against a reservoir solution consisting of 0.2 *M* ammonium sulfate, 0.1 *M* HEPES pH 7.5, 25% PEG 3350. Crystals of PCMB-derivatized Nb36-C85 were obtained by vapour diffusion against reservoirs containing either 0.1 *M* citric acid pH 3.5, 25% PEG 3350 (Nb36-C85-1) or 0.2 *M* sodium malonate pH 7.5, 20% PEG 3350 (Nb36-C85-2). Crystals were cryoprotected by transferring them stepwise into 35% PEG 3350 for Nb36-Nat1, Nb36-Nat2 and Nb36-C85-1, while Nb36-C85-2 crystals were transferred to mother liquor supplemented with 30% ethylene glycol, before being flash-cooled in liquid nitrogen.

### 2.5. Structure determination   

The data were processed with *XDS* and *XSCALE* (Kabsch, 2010 ▸). SAD phasing of the two PCMB derivatives was performed independently with *phenix.autosolve* (Terwilliger *et al.*, 2009 ▸). Subsequent density modification was performed with *phenix.autobuild* (Terwilliger *et al.*, 2008 ▸). Owing to the identification of multiple NCS operators in both cases, test-set reflections were selected in thin shells with *phenix.refine* (Afonine *et al.*, 2012 ▸) prior to automated model building with either *buccaneer_pipeline* (Cowtan, 2006 ▸) or *phenix.autobuild*. The resulting models were rebuilt in *Coot* (Emsley *et al.*, 2010 ▸) in an iterative manner and refined with *phenix.refine* until convergence using NCS restraints. A distance restraint of 2.3 Å between the SG atom of Cys85 and a connected Hg atom was present during refinement. A Nb molecule from the Nb36-C85-1 structure with the CDR regions deleted was used as a search model for molecular replacement with *phenix.phaser* (McCoy *et al.*, 2007 ▸) into the Nb36-Nat1 data collected from an underivatized crystal on beamline I04 at Diamond Light Source (DLS). The model was iteratively rebuilt in *Coot*, refined with *phenix.refine* and then used for molecular replacement into the Nb36-Nat2 data collected from an underivatized crystal on BioMAX at MAX IV; it was completed by iterative rebuilding and refinement. The quality of all structures was analysed with *MolProbity* (Chen *et al.*, 2010 ▸). Figures were prepared with *PyMOL* 1.8 (http://www.pymol.org).

### 2.6. Antigen-binding measurements   

Binding of native Nb36 and Hg-labelled Nb36-C85 to C5 was measured on an Octet RED biolayer interferometer (Pall ForteBio) in PBS buffer. Histidine-tagged Nbs were immobilized on Anti-Penta-HIS biosensors (Pall ForteBio) at a concentration of 2.5 µg ml^−1^ and amounted to approximately 0.2 nm saturation. Interaction with C5 was measured in a dilution series of antigen concentrations ranging from 62.5 to 2000 n*M* for 700 s. Subsequently, the dissociation was recorded for 1800 s. To account for baseline drift during the experiment, biosensors immobilized with Nbs dipped into PBS without C5 were measured in parallel and subsequently subtracted. Sensorgrams were processed using *ForteBio Data Analysis* 7.0 (Pall ForteBio) and data were globally fitted using nonlinear regression in *GraphPad Prism* 6 (GraphPad Software) with a goodness of fit (*R* ^2^) of 0.99 for both native Nb36 and PCMB-labelled Nb36-C85.

## 3. Results   

### 3.1. Selecting Nb36 against complement component C5   

**Figure 1:**
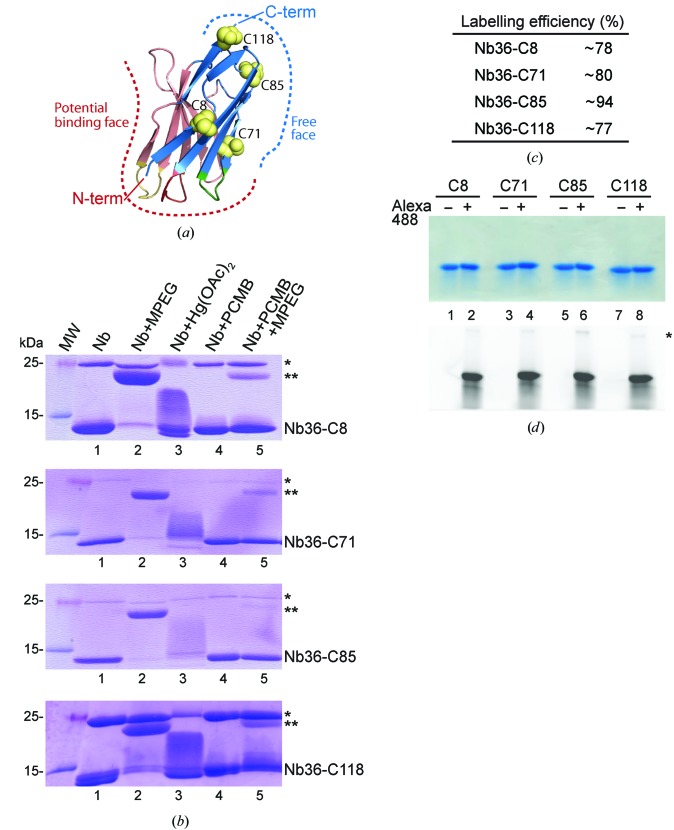
Site-specific labelling of Nbs. (*a*) Overview of a Nb structure (exemplified here by PDB entry 3p0g; Rasmussen *et al.*, 2011 ▸) with the face in red observed to contribute to antigen binding (potential binding face); the face in blue has never been observed to directly interact during antigen binding (free face). The positions of the four Ser-to-Cys mutations are indicated as yellow spheres on the free face of the Nb. (*b*) Nonreduced SDS–PAGE of the labelling reactions with the dimeric form of Nb36 (*) and MPEG-labelled Nb36 (**) indicated. (*c*) Quantification of labelling efficiency based on the MPEG–maleimide assay. (*d*) Alexa Fluor 488 labelling of the four cysteine-mutated Nbs. Nonreduced SDS–PAGE analysis of labelled and unlabelled Nbs (top) and the corresponding fluorescence scan of the gel (bottom). * indicates the fraction of dimeric Nb36 nonspecifically labelled with Alexa Fluor 488.

To identify Nb36 targeting C5, we immunized a llama and performed two rounds of phage display on the derived library. Finally, we performed ELISA and the identified clone was sequenced and subcloned into a bacterial expression vector. Next, by aligning 20 Nb–antigen structures from the PDB, we identified positions within the Nb framework for the introduction of a free cysteine that we predicted would not interfere with antigen binding. These chosen positions were also conserved among Nb families, ensuring the general applicability of our approach. Antigen binding can be mediated by the CDR loops which protrude from the N-terminal side of the Nbs. Alternatively, the CDR loops can adopt a conformation that occludes the face made up of β-strands *C*′′–*C*–*C*–*F*–*G*. In all of the Nb complexes examined the C-terminus and the *A*–*B*–*E*–*D* β-sheet face are freely available and do not engage in antigen binding (Fig. 1 ▸ *a*). For Nb36 we chose to substitute conserved serine residues and generate four cysteine mutants modified at one of the positions 8, 71, 85 or 118 (corresponding to positions 7, 70, 82b and 112 in the Kabat nomenclature). The native Nb36 and all variants with a single free cysteine were successfully expressed in *E. coli* and purified to homogeneity, producing milligram quantities.

### 3.2. Mercury and fluorophore labelling   

Purified Nb cysteine mutants (Nb36-C8, Nb36-C71, Nb36-C85 and Nb36-C118) were labelled with either mercury compounds or a fluorescent dye. For mercury labelling, Nbs were first exchanged into a nonreducing labelling buffer. This was necessary because standard reducing agents such as DTT and BME contain thiol groups that react with mercury compounds. When analysed by nonreducing SDS–PAGE all of the Nbs ran predominantly as monomers around 13 kDa, with various degrees of cysteine-mediated dimers appearing at 25 kDa (Fig. 1 ▸ *b*, lane 1). In particular, Nb36-C8 and Nb36-C118 had a higher tendency to form dimers compared with Nb36-C71 and Nb36-C85. To test the accessibility of the free cysteines in these Nb mutants, we labelled them with an MPEG moiety using maleimide chemistry. We were able to label the monomeric Nbs for all of the mutants, as seen by an ∼10 kDa shift in molecular weight (Fig. 1 ▸ *b*, lane 2), indicating that the free cysteine is accessible in all Nbs. We next examined whether it was possible to label the Nbs with either mercury(II) acetate or PCMB. Mercury(II) acetate treatment gave visible smeary bands (Fig. 1 ▸ *b*, lane 3), whereas PCMB modification of the Nbs was not clearly resolved by SDS–PAGE (Fig. 1 ▸ *b*, lane 4). To visualize the PCMB-labelling efficiency we post-treated with MPEG to detect the available free cysteines after PCMB labelling, and we clearly observed that most cysteines are inaccessible after PCMB treatment (Fig. 1 ▸ *b*, lane 5). Finally, we semi-quantified the PCMB-labelling efficiency by comparing the band intensities between MPEG-labelled Nb monomers and PCMB-labelled monomer bands post-treated with MPEG and found that the Nbs mutants were labelled with an efficiency of between 78 and 94% (Fig. 1 ▸ *c*).

Since Nbs are often used in high-resolution imaging and other experiments requiring fluorescent signals, we tested whether we could fluorescently label our Nb cysteine mutants with the Alexa Fluor 488 male­imide fluorescent dye. We again performed a buffer transfer to avoid the reaction of reducing-agent thiols with labelling reagents. The Coomassie Blue-stained SDS–PAGE showed single monomeric Nbs in all lanes (Fig. 1 ▸ *d*, top) and comparison with fluorescence imaging of the same gel (Fig. 1 ▸ *d*, bottom) revealed that fluorescence labelling was successful for all four Cys-mutant Nbs using a 1.5-fold molar excess of fluorescent dye.

In summary, we show that it is possible to introduce free cysteines at four different positions within the Nb framework and that we can label these efficiently. Nb36-C85 showed an overall high efficiency for both mercury and fluorescence labelling and a low tendency to form cysteine-mediated dimers, and we decided to continue our structural analyses using this version.

### 3.3. *De novo* phasing using incorporated mercury   

We were able to crystallize both native Nb36 and Nb36 Ser85Cys labelled with PCMB. For the derivatized protein we obtained crystals at two very different pH values of 3.5 (Nb36-C85-1) and 7.5 (Nb36-C85-2). To investigate the potential of the Hg-derivatized Nbs for rapid and automated structure determination without prior phase information, we analysed two SAD data sets exhibiting space groups *P*2_1_2_1_2_1_ (Nb36-C85-1) and *P*2_1_ (Nb36-C85-2). For the *P*2_1_2_1_2_1_ case, 720° of data were collected to a maximum resolution of 1.5 Å (see Table 1 ▸ for details). Analysis of the anomalous signal from the four consecutive 180° wedges indicated a significantly stronger signal in the first 180° of data compared with the subsequent 540° of data. The anomalous correlation between random half data sets according to *XSCALE* was 0.39 and 0.16 for the rotation ranges 1–180° and 180–720°, respectively. Importantly, even for the rotation range 540–720° the anomalous correlation was still 0.1 for the full resolution range with a clear anomalous signal in the resolution shell 2.74–2.54 Å, which exhibited an anomalous correlation of 0.18. For comparison the 1–180° data had an anomalous correlation of 0.19 in the 1.94–1.86 Å resolution shell. Remarkably, the *R*_meas_ values for the 1–180° and the 540–720° data were 0.054 and 0.055, respectively. Hence, in line with previous studies of Hg-substituted cysteine side chains (Ramagopal *et al.*, 2005 ▸), we observed a significant selective decay of the anomalous signal from Hg, whereas the overall data quality was preserved during the full rotation range.

**Figure 2:**
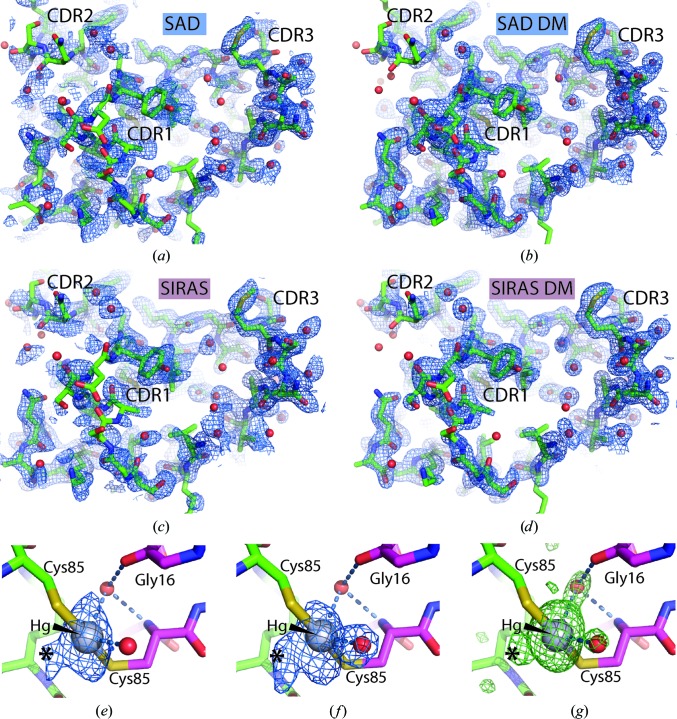
SAD- and SIRAS-based structure determination in *P*2_1_2_1_2_1_. (*a*) Electron density calculated from SAD phases based on the 1–180° data together with the final Nb36-C85-1 model. (*b*) As in (*a*) but after density modification. (*c*) The map calculated with SIRAS phases prepared using the 1–180° data as a derivative with anomalous signal and the 180–720° wedge as native data. (*d*) As in (*c*) but after density modification. (*a*)–(*d*) are contoured at 1.2σ. (*e*) Close-up of a Hg atom inserted between two NCS-related Cys85 side chains together with an anomalous map calculated with the anomalous differences from the 1–180° data and phases derived from the final model with Hg atoms and water molecules omitted. One minor site (marked with an asterisk) is already present in this wedge of data. (*f*) Anomalous map calculated from the anomalous differences in the 180–720° data revealing how the Hg site is splitting into one major and two minor sites. (*g*) Difference *mF*_o_− *DF*_c_ map calculated after omission of the Hg atoms and all water molecules from the final model based on the 1–180° data. The maps in (*e*) and (*f*) are contoured at 5σ, while the difference map in (*g*) is displayed at 3σ. Water molecules are shown as red spheres.

The pronounced decay of the anomalous signal inspired us to compare SAD phasing based on the 1–180° data with SIRAS phasing, in which the 180–720° data acted as the native data and the 1–180° data as a derivative with an anomalous signal. This strategy is also known as radiation-damage-induced phasing with anomalous scattering (RIPAS) and has previously been shown to strongly improve the resulting electron density obtained after phasing based on anomalous data with significant decay in the anomalous signal from Cys–Hg derivatives of the YjcF and YidA proteins (Ramagopal *et al.*, 2005 ▸). For site identification and phasing we used *phenix.autosol* and obtained figures of merit (FOMs) of 0.38 and 0.40 for the SAD and SIRAS scenarios, respectively. In the SAD case two major sites each with two minor sites located within 1.4–1.6 Å were modelled by *phenix.autosol*, whereas only the two major sites were modelled in the SIRAS phasing scenario. Despite the apparent modest differences in FOM for the scenarios, the quality of the SIRAS-based electron density prior to any density modification was clearly superior to that based on SAD phasing (Figs. 2 ▸ *a* and 2 ▸ *c*). Subsequent density modification in both cases resulted in easily interpretable maps (Figs. 2 ▸ *b* and 2 ▸ *d*), although the SIRAS-based map was still slightly superior. The SAD-based density-modified map based on the 1–180° data was used for automated model building and refinement with the *Buccaneer* pipeline, which traced the four molecules in the asymmetric unit almost completely. Subsequent refinement of the *Buccaneer* model with *phenix.refine* and inclusion of water molecules yielded a model with an *R*_free_ of 0.221. At this point the first manual rebuilding occurred including the incorporation of two Hg atoms, and a few cycles of iterative refinement and rebuilding resulted in a model with an *R*_free_ of 0.215.

As already indicated by the site identification in *phenix.autosolve*, the structure did not contain a single Hg atom at each free Cys85 introduced into the Nb. Instead, two Hg atoms each bridge two cysteine side chains from two NCS-related Nbs and no density could be assigned to the benzoate moiety. This implies that the benzoate was released at some point between the isolation of monomeric Hg-substituted Nb and cooling of the crystal. A water atom appears to adopt a third coordination position at both sites, and another water molecule 3.3 Å from the Hg atom is also present at both sites, but is more likely to be present owing to a nearby main-chain carbonyl group. When contoured above 6σ the Fourier map calculated with anomalous differences from the 1–180° data and phases calculated from the final model refined against the 1–180° data (with Hg atoms and water omitted) did not show much evidence of Hg subsites (Fig. 2 ▸ *e*). In contrast, when the same phases were used in combination with the anomalous differences from the 180–720° data, there was very clear evidence of two Hg subsites around each major site (Fig. 2 ▸ *f*). The strongest of these was separated from the major site by 3 Å and located at the position of a water molecule in the structure refined against the 1–180° data, whereas the anomalous density for the second minor site was continuous with that of the major site (Figs. 2 ▸ *e* and 2 ▸ *f*). The strongest subsite could in principle correspond to a Hg atom bound to only one cysteine, but there is no density supporting an alternative conformation of the nearest cysteine (Fig. 2 ▸ *g*). The strong subsite is therefore more likely to correspond to a Hg^2+^ ion released from both cysteine side chains. In contrast, the weakest subsite appears to stem from a fraction of molecules in which the Hg atom is only bound to one cysteine side chain since density supporting an alternative conformation of the nearby cysteine is present. A movement of Hg atoms induced by radiation damage was also noticed for the YidA protein (Ramagopal *et al.*, 2005 ▸).

**Figure 3:**
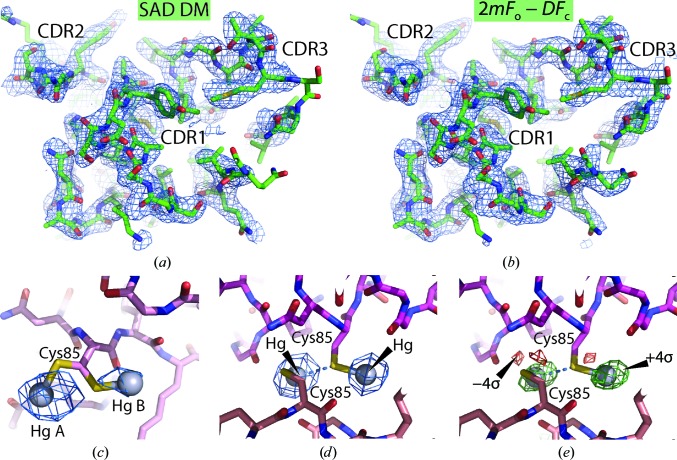
SAD-based structure determination in *P*2_1_. (*a*) Electron density calculated from density-modified SAD phases around the CDR loops of the final Nb36-C85-2 model. (*b*) As in (*a*) but displaying the final 2*mF*_o_ − *DF*_c_ map. (*a*) and (*b*) are contoured at 1.2σ. (*c*) Close-up of the double conformation of a Cys side chain bound to a Hg atom together with a map calculated from the anomalous differences and phases derived from the final model with Hg atoms omitted. The two sites were modelled with occupancies of 0.6 and 0.4, respectively, and the map is contoured at 6σ. (*d*) Anomalous map calculated as in (*c*) around two Cys85 side chains each bound to one Hg atom. The S atom of one of these cysteines apparently also forms a nonbonded interaction (dotted blue line) with the neighbouring Hg. The map is contoured at 16σ and comparison with (*c*) justifies that both Hg sites are modelled with full occupancy. (*e*) Difference *mF*_o_ − *DF*_c_ map from the final model contoured at 4σ (green) and −4σ (red) of the same two Hg atoms and cysteines further supporting this interpretation.

For the *P*2_1_ crystal 360° of data (Nb36-C85-2) were integrated to a *d*_max_ of 2.5 Å. The anomalous signal did not extend beyond the 3.5–3.7 Å resolution shell, which had an anomalous correlation between random half data sets of 11% according to *XSCALE*. Owing to the low symmetry, 180° of spindle rotation was required to obtain a complete set of anomalous differences. Since only a minor difference between the anomalous signal in the two segments 1–180° and 180–360° was observed we did not pursue SIRAS phasing in this case, but instead performed SAD phasing based on all 360° of data. This resulted in a set of phases with an overall FOM of 0.34 based on 19 sites in eight groups. As also observed in the *P*2_1_2_1_2_1_ case, some of the *phenix.autosol* minor sites were within 2 Å of the major sites, reflecting their role in modelling of a nonspherical major site, whereas sites separated by 5–7 Å reflected more distinct sites that were also mirrored in the refined model (see below). Density modification with *phenix.autobuild* revealed clear electron density for multiple Nbs in the asymmetric units (Fig. 3 ▸ *a*). Upon automated model building and refinement with *phenix.autobuild*, the majority of the nine molecules were traced and the resulting model, which was 81% complete compared with the final model, had an *R*_free_ of 0.332. OMIT density for the tenth Nb molecule was clearly visible in a 2*mF*_o_ − *DF*_c_ map, but an MR search model derived from the *P*2_1_2_1_2_1_ structure could not be placed correctly with *phenix.phaser* using either a standard or a phased translation function and the tenth NCS copy was therefore docked manually. After iterative model building and refinement, a final model with an *R*_free_ of 0.275 was obtained. In this model water molecules were not included, as their inclusion did not decrease the *R*_free_. The Nb molecule that was placed manually appears to have a slight rotational freedom within the crystal packing, since the CDR pole of the Nb has very poor electron density, whereas the opposite end containing Cys85 has much better defined density.

In the final *P*2_1_ model (Fig. 3 ▸ *b*), which includes nine Hg atoms, all ten Cys85 side chains are bound to Hg but in four different manners. Two of the Hg atoms are each inserted between two side chains, as also observed in the *P*2_1_2_1_2_1_ crystal form. Another pair of Hg atoms with modelled occupancies of 0.6 and 0.4 are bonded to two alternative side-chain conformations of the same cysteine (Fig. 3 ▸ *c*), which gives rise to a separation of 5.5 Å between the two Hg sites. This leaves five Hg atoms that are bound only to a single cysteine. Interestingly, amongst these there are two very similar arrangements in which two Hg atoms, each bound to a single cysteine side chain, are located 5.3 Å from each other (Figs. 3 ▸ *d* and 3 ▸ *e*). In both cases one of the two Hg atoms appears to neighbour a nonbonded cysteine SG atom ∼3.1 Å away in addition to the SG atom that it is directly bonded to. As for the *P*2_1_2_1_2_1_ Hg sites there is little or no density that may be readily attributed to a benzoic acid moiety of PCMB.

### 3.4. Structure of Nb36   

**Figure 4:**
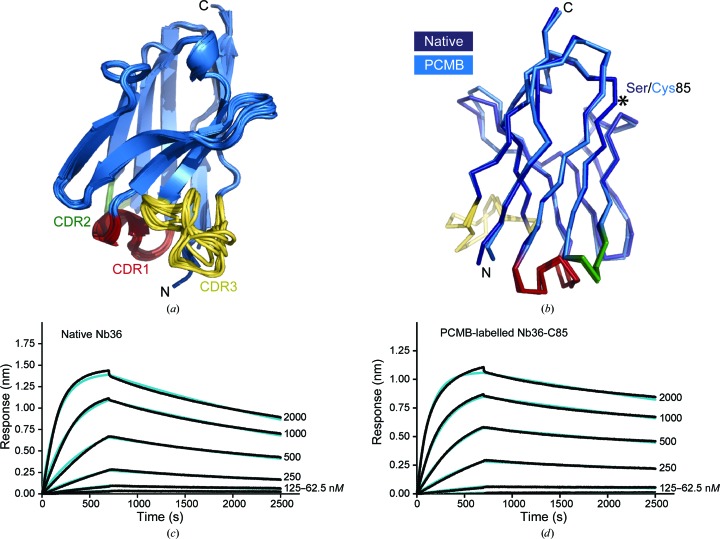
The structure and antigen binding is not perturbed by the Ser85Cys mutation. (*a*) Overlay of the 16 different Nb36 molecules present in the four different crystals, with CDR1, CDR2 and CDR3 coloured red, green and yellow, respectively. (*b*) Structural superposition of native Nb36-Nat1 (light blue) and PCMB-labelled Nb36-C85-1 (dark blue). (*c*, *d*) Binding of the C5 antigen to immobilized native Nb36 or PCMB-labelled Nb36-C85 measured by biolayer interferometry. The experimental association and dissociation curves are shown in black and the fitted curves are shown in blue.

Nb36 shows the classical two-layered structure built of four-stranded and five-stranded antiparallel β-sheets connected by loops (Fig. 4 ▸ *a*). The three CDR loops protrude from the N-terminal end of this compact Ig fold and form the antigen-binding surface. Comparing and overlaying the 16 different Nb36 molecules from the four different crystals reveals that the Nb framework is structurally similar and the variations are within the flexible CDRs and especially the longer CDR3 loop (Fig. 4 ▸ *a*). Since we obtained the crystal structure of both native Nb36 and PCMB-labelled Nb36-C85 we can compare their structures. The superposition shows that the non-CDR framework residues are not structurally affected by mutating Ser85 to Cys (Fig. 4 ▸ *b*). Next, we tested whether Hg labelling compromises binding to the C5 antigen. We performed biolayer interferometry experiments in which we immobilized either native Nb36 or PCMB-labelled Nb36-C85 on Anti-HIS biosensors and measured binding at different concentrations of C5. These experiments suggested that native Nb36 binds C5 with an equilibrium dissociation constant (*K*_d_) of ∼86 n*M* (Fig. 4 ▸ *c*) and PCMB-labelled Nb36-C85 binds C5 with a *K*_d_ of ∼37 n*M* (Fig. 4 ▸ *d*). Overall, our studies show that it is possible to introduce free cysteines in the Nb framework and express, purify and label these with a fluorescent group or Hg. For one selected variant of the C5-binding Nb we show that Hg derivatization does not compromise antigen binding or influence the framework structure. Importantly, our data provide a route for simple experimental phasing using Hg-derivatized Nbs.

## 4. Discussion   

It was our expectation that the crystallization of a monomeric PCMB-derivatized Nb would reveal structures with a Hg atom inserted between the benzoate moiety and the side chain of Cys85. In the two PCMB structures we have modelled a total of 11 Hg atoms; four of these were clearly bridging two cysteines, but the remaining seven did not display density that could be attributed to the benzoate fragment. This suggests a high tendency for benzoate release, possibly by a mechanism reminiscent of the protonolysis of organomercurial compounds catalysed by the MerB enzyme, which can accelerate protonolysis by up to 10^7^ (Parks *et al.*, 2009 ▸). Models of the MerB reaction mechanism suggest that the release of the organic substituent is catalysed through its protonation by an aspartic side chain that acts as a proton shuttle during transfer of the proton from one of the reacting cysteine –SH groups to the C atom bound to Hg (Parks *et al.*, 2009 ▸), here the benzoate C4 atom. Whether this mechanism of benzoate release is relevant in our Nb36-C85 PCMB crystallization experiments cannot be decided upon, especially since we do not have a structure of the intermediate from which the benzoate is released. There are several deposited structures in the PDB containing intact PCMB (residue identifier MBO) in which the CysS–Hg–benzoate substructure is modelled. Intriguingly, there are also two entries, PDB entries 5ec5 (Podobnik *et al.*, 2016 ▸) and 1naq (Arnesano *et al.*, 2003 ▸), in which PCMB has been used in which some of the Hg sites have the benzoate modelled while other sites have only the Hg bound to the cysteine. Hence, the release of benzoate observed in our structures appears to be a general feature of this reagent. Although unintentional, it may have given rise to new crystal forms as our crystals of non­derivatized Nb36 exhibit *C*2 symmetry in contrast to *P*2_1_ or *P*2_1_2_1_2_1_ symmetry for the PCMB-derivatized Nb, and in both cases there are Hg-bridged Nb dimers. Methane and ethane are released very slowly from organomercurials by protonolysis (Begley *et al.*, 1986 ▸), suggesting that the use of mercury compounds such as CH_3_HgCl and CH_3_CH_2_HgCl may minimize the observed unintentional liberation of the organic group during crystallo­genesis and storage.

It is interesting that in our *P*2_1_ case, which has ten Hg-derivatized Nbs in the asymmetric unit, there are two different arrangements that both lead to a separation of two Hg sites by ∼5.5 Å. This may be utilized in heavy-atom search procedures, where using a pair of Hg atoms separated by 5.5 Å as a model of such a super-Hg site may be beneficial in the same manner as the use of the known geometry of disulfide bridges by *SHELXD* (Sheldrick, 2008 ▸). Our results demonstrate that SAD-based structure determination of a 14 kDa Nb with either one or half a Hg atom bound is straightforward, but the potential of Hg-substituted Nbs in crystallography goes far beyond this. For any crystal containing a Nb–antigen complex experimental phases can most likely be obtained by using a Hg-substituted Nb for co-crystallization. For very large antigens, the anomalous signal may be considerably enhanced by reacting the Nb with the four-mercury cluster tetrakis(acetoxymercuri)methane, which is a classical compound for the phasing of large structures by isomorphous substitution (O’Halloran *et al.*, 1987 ▸; Andersen *et al.*, 1995 ▸).

The introduction of cysteines and subsequent conjugation with malemides opens a range of possible applications beyond phasing in protein crystallography. Their high specificity and small size make Nbs especially useful for imaging. Nbs have previously been labelled with fluorophores using NHS esters (Ries *et al.*, 2012 ▸) and by maleimides (Pleiner *et al.*, 2015 ▸) that react with introduced cysteines as described in the current study. Compared with nonspecific lysine NHS ester labelling, site-specific labelling with maleimide fluorophores resulted in less background and better paratope preservation (Pleiner *et al.*, 2015 ▸). In non-invasive *in vivo* imaging, Nbs have been applied in radionuclide-based techniques including position emission tomography (Vosjan *et al.*, 2011 ▸) and single-photon emission computed tomography (Huang *et al.*, 2008 ▸). Here, the chelating agents that bind the radionuclide were conjugated to lysine residues or through a C-terminal His tag and the introduction of free cysteines may allow alternative conjugation chemistries (George *et al.*, 1995 ▸). The ability to site-specifically conjugate cytotoxic molecules to Nbs and antibodies is desired during the generation of antibody–drug conjugates. Currently licensed antibody–drug conjugates are produced by nonspecific labelling of lysine residues, resulting in a mixture of heterogeneous molecules with different numbers of cytotoxic molecules conjugated to each antibody (Diamantis & Banerji, 2016 ▸). Precise control of the number of cytotoxic molecules that are conjugated by labelling though introduced cysteine residues would eliminate the heterogeneity and increase the therapeutic potential, as has already been shown for IgG antibodies (Panowski *et al.*, 2014 ▸). Likewise, the introduction of cysteines may allow site-specific and controlled PEGylation to increase stability and extend the half-life of Nbs in a therapeutic setting.

In conclusion, we show that by introducing Hg-reactive cysteines in the Nb framework we are able to routinely obtain high-quality experimental phases from anomalous data. Here, we have used this for structure determination of the derivatized Nb, but this approach will also provide rapid access to the structure determination of larger protein complexes containing Nbs. In a wider perspective, our results also offer a route to site-specific Nb modifications, which will be highly beneficial in imaging and drug-development applications.

## Supplementary Material

PDB reference: Nb36-Nat1, 5nlu

PDB reference: Nb36-Nat2, 5nlw

PDB reference: Nb36-C85-1, 5nm0

PDB reference: Nb36-C85-2, 5nml

## Figures and Tables

**Table 1:** Data-collection and refinement statistics Values in parentheses are for the highest resolution shell.

	Nb36-Nat1	Nb36-Nat2	Nb36-C85-1	Nb36-C85-2
PDB code	5nlu	5nlw	5nm0	5nml
Beamline	I04, DLS	BioMAX, MAX IV	P13, PETRA	ID23, ESRF
Wavelength (Å)	0.9686	0.9796	1.008	1.008
Resolution range (Å)	29.3–1.193 (1.236–1.193)	28.81–1.499 (1.553–1.499)	71.25–1.50 (1.554–1.500)	45.41–2.50 (2.589–2.500)
Space group	*C*2	*C*2	*P*2_1_2_1_2_1_	*P*2_1_
Unit-cell parameters				
*a* (Å)	102.56	101.86	37.61	83.96
*b* (Å)	29.27	30.04	93.07	99.11
*c* (Å)	33.13	32.89	142.51	85.41
α (°)	90	90	90	90
β (°)	97.011	90.837	90	105.772
γ (°)	90	90	90	90
Total reflections	203833 (10799)	51526 (3091)	521268 (48924)	268259 (11613)
Unique reflections	30668 (2451)	15233 (1123)	81018 (7976)	43707 (3010)
Multiplicity	6.6 (4.4)	3.4 (2.7)	6.4 (6.1)	6.1 (3.9)
Completeness (%)	97.67 (78.67)	93.81 (70.34)	99.80 (99.89)	93.59 (64.95)
Mean *I*/σ(*I*)	10.65 (1.15)	10.26 (1.06)	17.44 (1.57)	14.38 (0.80)
Wilson *B* factor (Å^2^)	15.87	25.95	22.30	67.63
*R*_merge_	0.08345 (1.022)	0.05309 (0.7674)	0.05807 (1.111)	0.08839 (1.5)
*R*_meas_	0.09057 (1.153)	0.06285 (0.936)	0.06328 (1.214)	0.09642 (1.743)
*R*_p.i.m._	0.03457 (0.5135)	0.03297 (0.5247)	0.02482 (0.4852)	0.0379 (0.8658)
CC_1/2_	0.998 (0.496)	0.998 (0.399)	0.999 (0.641)	0.998 (0.185)
CC*	0.999 (0.814)	1 (0.755)	1 (0.884)	1 (0.559)
Reflections used in refinement	30641 (2445)	15222 (1122)	81007 (7975)	43704 (3010)
Reflections used for *R*_free_	1998 (159)	1529 (111)	2018 (202)	2014 (132)
*R*_work_	0.1750 (0.4562)	0.1992 (0.3669)	0.1886 (0.3065)	0.2239 (0.3770)
*R*_free_	0.1979 (0.5825)	0.2484 (0.3749)	0.2114 (0.3066)	0.2617 (0.3639)
CC_work_	0.970 (0.677)	0.963 (0.666)	0.955 (0.817)	0.932 (0.400)
CC_free_	0.976 (0.590)	0.934 (0.663)	0.949 (0.764)	0.928 (0.342)
No. of non-H atoms				
Total	1046	965	3864	8487
Protein	902	887	3483	8462
Ligands	5	5	2	25
Solvent	139	73	379	
Protein residues	118	117	462	1128
R.m.s.d., bonds (Å)	0.008	0.002	0.004	0.003
R.m.s.d., angles (°)	1.03	0.49	0.78	0.70
Ramachandran favoured (%)	99.14	97.39	96.90	97.81
Ramachandran allowed (%)	0.86	2.61	2.21	1.73
Ramachandran outliers (%)	0.00	0.00	0.88	0.46
Rotamer outliers (%)	3.00	2.04	2.11	3.18
Clashscore	4.43	1.69	4.18	3.69
Average *B* factor (Å^2^)				
Overall	21.66	40.15	31.33	84.93
Protein	20.47	40.00	30.68	84.92
Ligands	20.31	29.45	38.53	86.82
Solvent	29.37	42.72	37.28	
No. of TLS groups	1	1	4	10
